# The morphology and bridging of the sella turcica in adult orthodontic patients

**DOI:** 10.1186/s12903-018-0499-1

**Published:** 2018-03-16

**Authors:** Gunjan Kumar Shrestha, Prabhat Ranjan Pokharel, Rajesh Gyawali, Bhushan Bhattarai, Jamal Giri

**Affiliations:** 1 Department of Orthodontics, M.B. Kedia Dental College, Rajat Jayanti chowk, Birgunj, 44300 Nepal; 2 Department of Orthodontics, College of Dental Surgery, B.P. Koirala Institute of Health Sciences, Dharan, 56700 Nepal; 3 Department of Orthodontics, Nobel Medical College, Biratnagar, 56600 Nepal

**Keywords:** Bridging, Morphology, Sella turcica, Size, Skeletal patterns

## Abstract

**Background:**

The purpose of this study was to determine and compare the shapes, sizes, and bridging of the sella turcica in patients with different skeletal patterns and genders.

**Methods:**

It was a cross-sectional comparative study. The samples were divided into three groups according to the skeletal pattern viz. Class I, Class II and Class III, and each group consisted of 40 samples (20 males and 20 females). The lateral cephalograms were traced and the sella turcica was assessed for its size, shape, and bridging.

**Results:**

The mean length, anteroposterior diameter and depth of sella turcica were 8.13 ± 2.03 mm, 9.60 ± 1.43 mm and 6.40 ± 1.21 mm respectively. The mean length of sella turcica was 7.91 ± 1.52 mm in Class I, 7.32 ± 1.62 mm in Class II and 9.16 ± .2.42 in Class III skeletal pattern; anteroposterior diameter was 9.30 ± 1.02 mm in Class I, 9.15 ± 1.28 mm in Class II and 10.35 ± 1.64 mm in Class III skeletal pattern; and the depth was 6.40 ± 0.92 mm in Class I, 6.07 ± 1.01 mm in Class II and 6.74 ± .1.54 mm in Class III skeletal pattern. There were significant differences in length and anteroposterior diameter and sella turcica between Class I, Class II and Class III skeletal patterns (*p* = 0.01), (*p* = 0.01) respectively. There was no significant difference in size of sella turcica between different genders and age groups. Sixty percent of the patients studied had normal Sella morphology. Partial Sella turcica bridging and Sella turcica bridging was seen in this study in 23.33% and 11.67% of patients respectively.

**Conclusion:**

Sixty percent of the patients had normal sella turcica. There were significant differences in lengths and anteroposterior diameters among Class I, Class II and Class III patients. The larger size was present in skeletal Class III patients.

## Background

Lateral cephalogram is a routinely used radiograph in Orthodontics for diagnosis, treatment planning and assessment of skeletal maturation [1]. Sella turcica is one of the landmarks that is commonly used in Cephalometry [1]. The sella turcica is a saddle-shaped bony structure in which anterior wall is formed by tuberculum sellae and posterior wall is formed by dorsum sellae. The pituitary fossa is surrounded by two anterior and two posterior clinoid processes [2]. The anterior and posterior walls of the Sella turcica have different developmental origin where the anterior wall develops from the neural crest cells and the posterior wall develops from paraxial mesoderm under the direct influence of notochord [3, 4].

The abnormalities in the sella turcica/pituitary gland can be associated with abnormalities within frontonasal, maxillary, palatal and mandibular developmental fields which forms the craniofacial regions [3]. An abnormal size of Sella turcica in Lateral Cephalograms can be seen conditions such as hyperprolactinemia [5], pituitary adenoma [6] Williams syndrome [7] and sometimes an enlarged sella turcica size may point out to an undiagnosed pathology or a condition [6]. The shape of the Sella may also be abnormal in different pathological conditions and syndromes, such as Down syndrome [8], Williams’s syndrome [7]. When the size of Sella turcica was considered with the different skeletal patterns, a larger size of Sella turcica was seen in skeletal Class III subjects [9–11] while smaller diameter was present in Class II subjects [9, 10]. Sella turcica bridge is a fusion of the anterior and posterior clinoid processes [2]. In Class III skeletal pattern [2, 12–14], dental anomalies [15] and dental transposition [16] higher incidence of Sella turcica bridging were found. The information on the size, shape, and degree of bridging of sella turcica in Nepali population is absent in the published literature. Morphological variations of Sella turcica can be seen in individuals to individuals, and the building the standards norms will help in the process of dismissing any anomaly in this vital region [9].

The objective of the study was to determine and compare the average shape, size, and bridging of sella turcica using lateral cephalogram in patients with different skeletal patterns and genders.

## Methods

It was a cross-sectional comparative study. The samples for the study were selected from the patients attending to the Department of Orthodontics and Dentofacial Orthopaedics OPD, B.P. Koirala Institute of Health Sciences. The ethical clearance was obtained from Institutional Ethical Review Board of BPKIHS, Dharan (Code no: IERB/361/014). Written Informed Consent was obtained from all patients in this study.

The patients in the study were Nepali citizens from 18 to 30 years age group having the clearest reproduction of sella turcica in lateral cephalogram without any craniofacial deformities, craniofacial syndromes, dental anomalies and medical conditions which have been reported to cause a change in shape, size and bridging of sella turcica. The sample size calculation was done using the formula given by Pocock [17]:

$$ \mathrm{Sample}\ \mathrm{size}=\mathrm{f}\ \left(\upalpha, \upbeta \right)\times 2{\upsigma}^2/{\left({\upmu}_1-{\upmu}_2\right)}^2 $$

$$ {\displaystyle \begin{array}{c}\mathrm{Taking}\ \upalpha =0.05\\ {}\upbeta =0.2\\ {}\upsigma =1.327\ \mathrm{Alkofide}\ \mathrm{EA}\ (2007)\\ {}{\upmu}_1-{\upmu}_2=0.9\\ {}\mathrm{Sample}\ \mathrm{size}=34.13\ \left(\mathrm{approximately}\ 35\right)\end{array}} $$

Hence 40 samples were taken in each group (20 males and 20 females). The total sample size in the study was 120.

Classification of skeletal type into Class I, Class II or Class III was based on the ANB, beta angle and W angle. The patients were grouped into a particular skeletal class when at least two out of three of the parameters defined it as that type of skeletal class.

**For ANB angle** [9, 18–20]**:**

**Class I:** ANB angle 0-4 degree.

**Class II:** ANB angle> 4.

**Class III:** ANB angle < 0.

**For Beta angle** [21]**:**

**Class I:** Beta angle 27° and 35°.

**Class II:** Beta angle < 27°.

**Class III:** Beta angle > 35°.

**For W angle** [22]**:**

**Class I:** W angle 51° and 56°.

**Class II:** W angle < 51°.

**Class III:** W angle > 56°.

The Gendex Orthoralix 9200 DDE machine was used for all lateral cephalograms. All radiographs were taken by a single trained radiographic technician. All films were laser printed on 10 × 12 in. Kodak dry view^Tm^. The mid-sagittal enlargement was 110%, and all linear measurements were corrected for magnification differences before the statistical analysis.

The sella turcica on each cephalometric radiograph was traced on 0.003 in. thick acetate matte tracing paper under optimal illumination. The shape and configuration of the sella turcica were drawn and only one observer was involved to determine the shape, size, and bridging of sella turcica.

**The shape of the sella turcica** [23]**:** Sella turica morphology was grouped according to the definitions of Axelsson et al. [23]^:^ as normal, oblique anterior wall, double contour of the floor, Sella turcica bridge, irregular notching in the posterior wall of dorsum sellae, pyramidal shape of dorsum sellae.

**Fig. 1:**
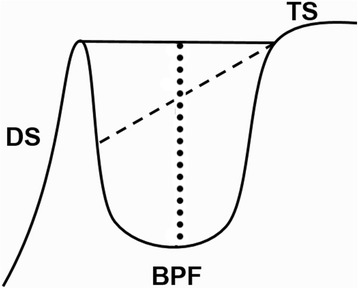
Normal sella turcica morphology and reference lines used for measuring sella size: TS, tuberculum sella; DS, dorsum sella; BPF, base of the pituitary fossa; black line, length of sella; dashed line, Antero-posterior diameter of sella; dotted line, depth of sella

**Size of the sella turcica:** The linear dimensions of sella turcica was measured by using the methods of Silverman [24] as cited by Axelsson et al. [23] as shown in (Fig. 1). All the reference lines drawn in this study were located in the midsagittal plane.

**Length of sella turcica** - The distance from the tuberculum sella to the tip of the dorsum sella was measured.

**The depth of the sella turcica-** A perpendicular from the above line to the deepest point on the floor was constructed and measured.

**Anteroposterior diameter of sella turcica-** from the tuberculum sella to the furthest point on the posterior inner wall of the fossa was drawn and measured.

The measurements were done by digital calipers (Mitutoyo, Japan) which measures up to 0.01 mm.

### Sella turcica bridging [15]

The sella turcica bridging was classified according to Leonardi R et al. [15]. The grading was based on length and anteroposterior diameter of Sella turcica which was as follows:

Class I (No calcification): Length of Sella turcica > 3/4th of the greatest anteroposterior diameter of Sella turcica.

Class II (Partial calcification): Length of Sella turcica ≤3/4th of the greatest anteroposterior diameter of Sella turcica.

Class III (Complete calcification): radiographically observable or identifiable diaphragma sella.

Data were analyzed using Statistical Package for Social Science Version 11.5 (SPSS Inc. Chicago, Illinois, USA). For inferential statistics, while comparing gender with the size of sella turcica Mann Whitney U test was used, and while comparing skeletal patterns with the size of sella turcica Independent samples Kruskal-Wallis test with Post Hoc analysis was used. Pearson’s correlation was used for comparison of the size of sella turica and age. Chi-square test was used for intergroup comparison. Bland-Altman’s Method was used to test reliability for SNA, SNB, ANB, W angle, Beta angle, Length, AP diameter, and Depth.. Kappa value was used to test reliability for the skeletal pattern, Bridging, and shape.

## Results

Kolmogorov-Smirnov and Shapiro-Wilk test was used to test the normality of distribution of the variables and the variables were found to be not normal in distribution (Table 1).

**Table 1:** Test for normality

	Kolmogorov-Smirnov			Shapiro-Wilk		
	Statistic	df	Sig.	Statistic	df	Sig.
Length	.117	120	.000	.942	120	.000
AP diameter	.093	120	.013	.933	120	.000
Depth	.073	120	.183	.944	120	.000
Age	.225	120	.000	.900	120	.000

### Size of the Sella turcica

The mean length, anteroposterior and depth of sella turcica were 8.13 ± 2.03 mm, 9.60 ± 1.43 mm and 6.40 ± 1.21 mm respectively (Table 2). There were no significant differences in size of sella turcica when compared with age (Table 3) and gender (Table 4). Significant differences in length and anteroposterior diameter and sella turcica were found between Class I, Class II and Class III skeletal patterns (*p* **=** 0.01**)**, (*p* **=** 0.01**)** respectively (Table 5). In post hoc analysis with Pairwise comparison of sella turcica size with skeletal class, differences in the mean length and anteroposterior diameter were significantly larger in Class III patients in comparison to Class II patients. (Table 5). The anteroposterior diameter was significantly larger in Class III patients in comparison to Class I patients (Table 5).

**Table 2:** Dimensions of sella turcica with Mean and Standard deviation

Parameters	Mean ± Standard deviation	Range
Length in mm	8.13 ± 2.03	3.90-15.39
Anetroposterior diameter in mm	9.60 ± 1.43	6.41-15.98
Depth in mm	6.40 ± 1.21	3.73-12.61

**Table 3:** Comparison of sella turcica size with age

Parameters	Pearson correlation	Significance
Age and Length	0.040	0.660
Age and AP diameter	0.015	0.873
Age and Depth	−0.156	0.088

**Table 4:** Comparison of size of sella turcica between genders

	Gender	*N*	Mean ± Standard deviation	*P* value
Length	Male	60	8.27 ± 2.14	0.679
	Female	60	8.00 ± 1.92	
Diameter	Male	60	9.56 ± 1.60	0.251
	Female	60	9.65 ± 1.25	
Depth	Male	60	6.18 ± 1.10	0.087
	Female	60	6.62 ± 1.29

**Table 5:** Comparison of size of sella turcica in different skeletal patterns

	Skeletal class	Number	Mean ± Standard deviation	*P* value	Pairwise comparison	*P* value	Mean difference
Length	Class I	40	7.91 ± 1.52	0.01	Class I- Class II	0.149	0.587
	Class II	40	7.32 ± 1.62		Class I- Class III	0.020	−1.253
	Class III	40	9.16 ± 2.42		Class II- Class III	< 0.001	−1.841
AP diameter	Class I	40	9.3 ± 1.02	0.01	Class I- Class II	0.751	0.151
	Class II	40	9.15 ± 1.28		Class I- Class III	0.003	−1.040
	Class III	40	10.35 ± 1.64		Class II- Class III	0.001	−1.191
Depth	Class I	40	6.40 ± 0.92	0.12			
	Class II	40	6.07 ± 1.01				
	Class III	40	6.74 ± 1.54				

### Shape of the Sella turcica

**Fig. 2:**
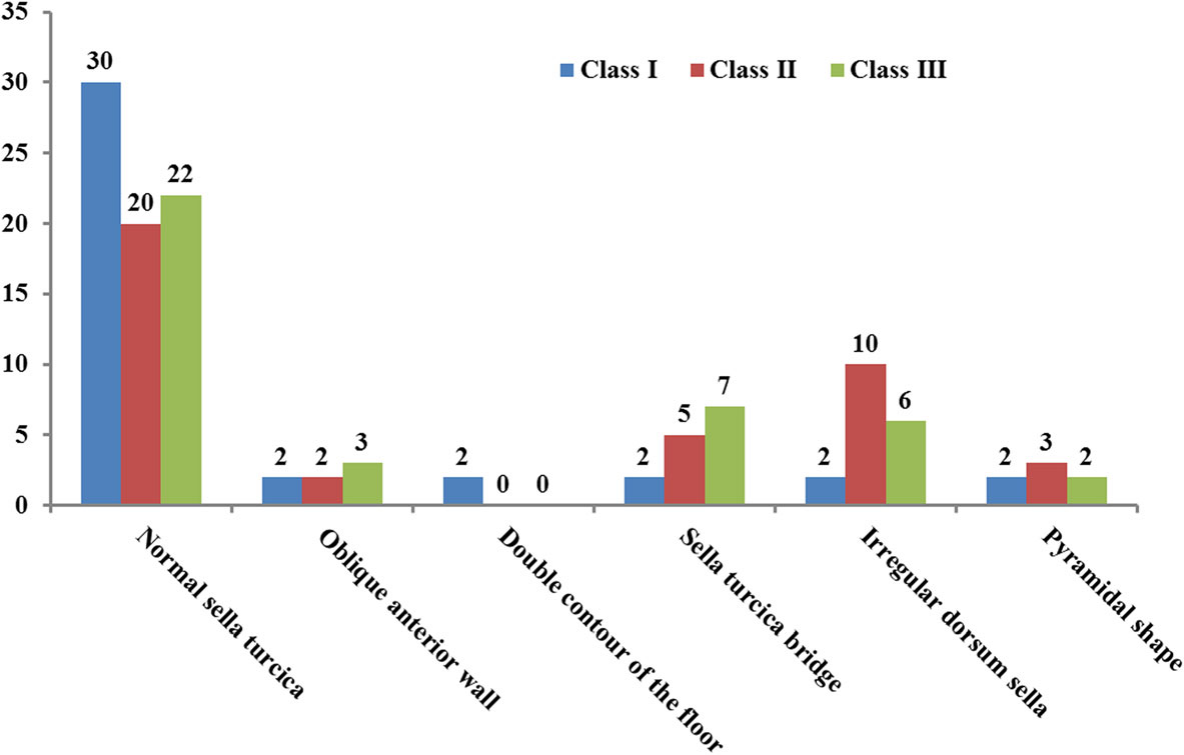
Shapes of sella turcica in different skeletal patterns

**Fig. 3:**
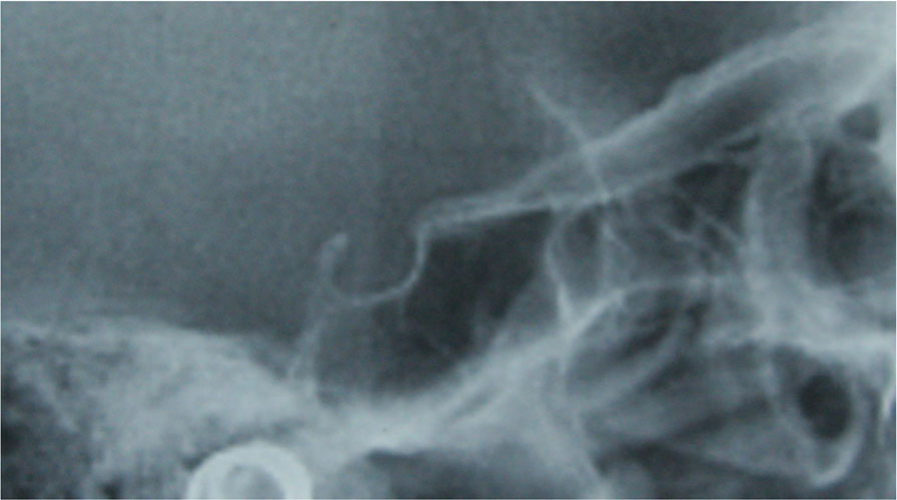
Normal morphology

**Fig. 4:**
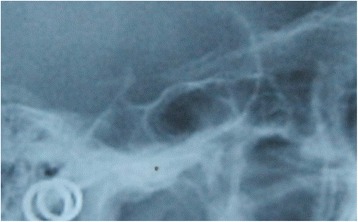
Oblique anterior wall

**Fig. 5:**
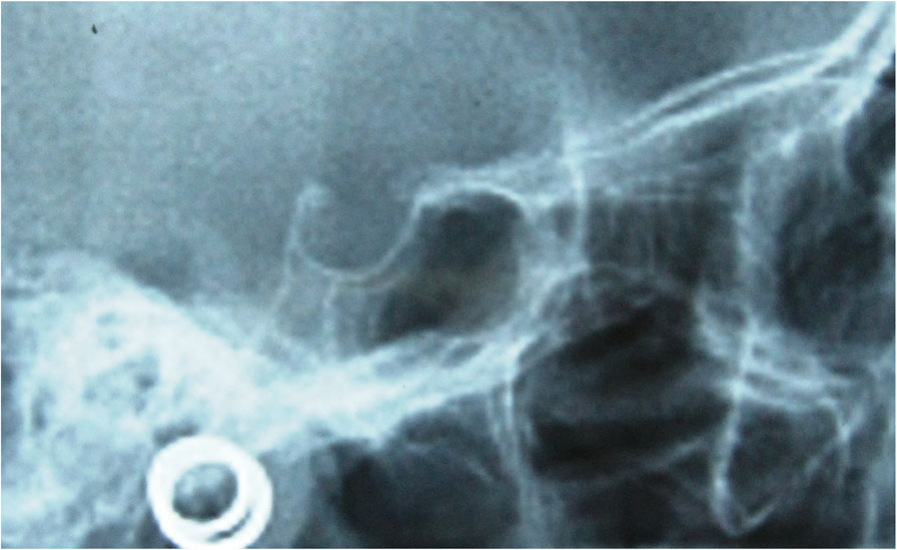
Double contour of the floor

**Fig. 6:**
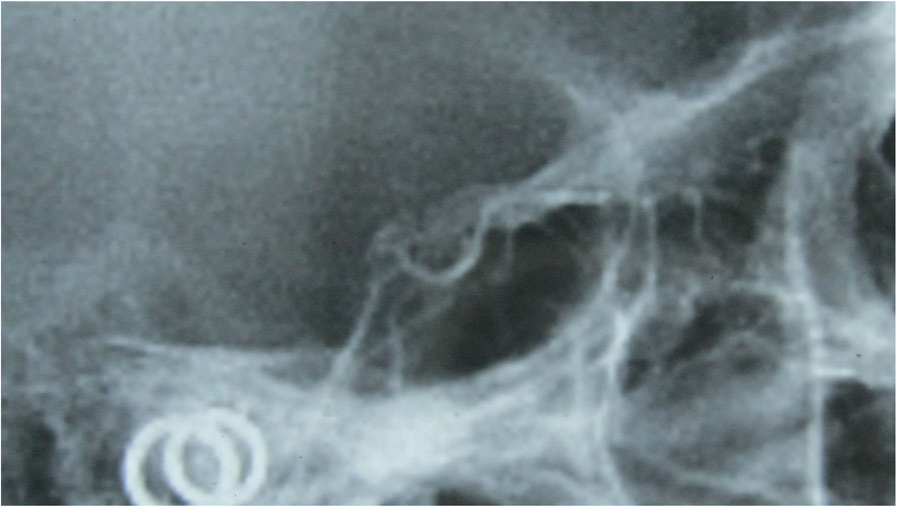
Sella turcica bridging

**Fig. 7:**
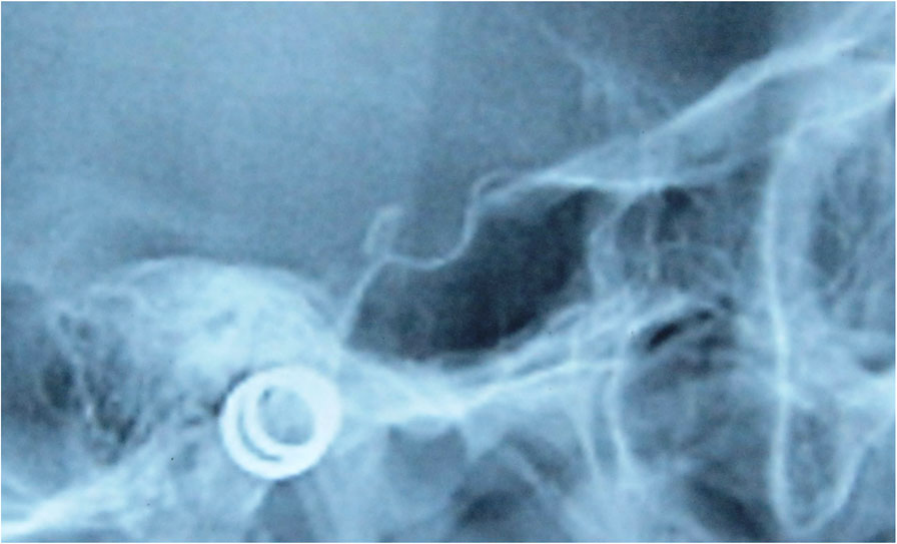
Irregularity(notching) in posterior wall of dorsum sellae

**Fig. 8:**
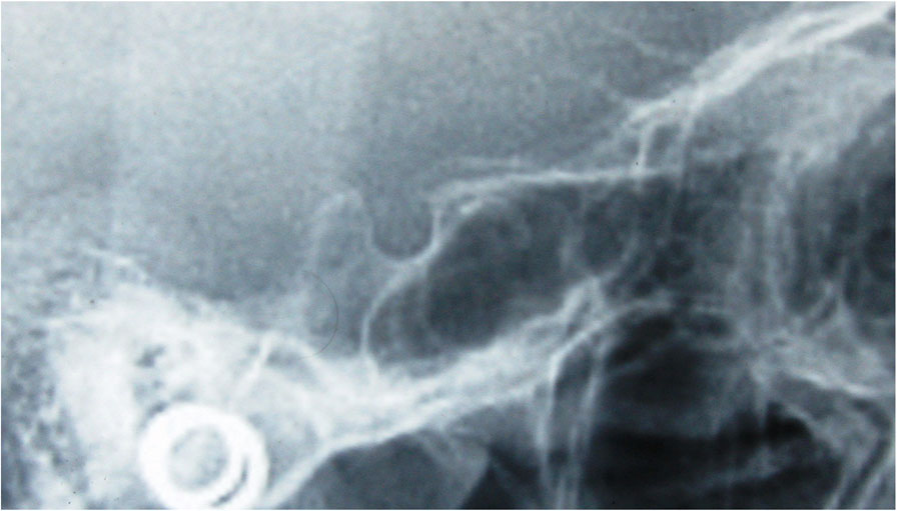
Pyramidal shape of dorsum sellae

The morphology of the sella turcica appeared to be normal in the majority of subjects (60%). The variation in the morphology of sella turcica was found in 40% of individuals (Table 6). There was no significant difference between the shapes of sella turcica between the genders (Table 6) and skeletal classes (Fig. 2). All the five variations of the morphology of sella turcica along with the normal morphology as given by Axelsson et al. [23] was found in this study (Figs. 3, 4, 5, 6, 7 and 8).

**Table 6:** Comparison of Shape of Sella turcica with gender

Shape	Total	Gender		*P* value
		Male	Female	
Normal sella turcica	72	32 (53.33℅)	40 (66.67℅)	0.187
Oblique anterior wall	7	5 (8.33℅)	2(3.33℅)	
Double contour of the floor	2	0	2 (3.33℅)	
Sella turcica bridge	14	9 (15℅)	5 (8.33℅)	
Irregular dorsum sella	18	9 (15℅)	9(15℅)	
Pyramidal shape	7	5 (8.33℅)	2 (3.33℅)	
Total	120	60	60

### Sella turcica bridging

No calcification was seen in 65% (*N* = 78), partial calcification was observed in 23.33% (*N* = 28) and complete calcification was seen in 11.67% (*N* = 14) of patients in this study (Table 7). The sella turcica bridging was not statistically significant among the three skeletal patterns, but complete calcification was seen more in Class III patients in comparison to Class I and Class II (Table 7).

**Table 7:** Comparison of Sella turcica bridging with skeletal patterns

Skeletal	Total	Sella turcica bridging			*P* value
		No calcification	Partial calcification	Complete calcification	
Class I	40	30 (75%)	8 (20%)	2(5%)	0.109
Class II	40	23(57.5%)	12(30%)	5(12.5%)	
Class III	40	25(62.5%)	8(20%)	7(17.5%)	
Total	120	78(65%)	28(23.33%)	14(11.67%)	

### Reliability of measurements

The reliability of measurements was determined by choosing 25% of lateral cephalometric radiographs at random, 30 lateral cephalograms (10 from each group) were retracted under identical conditions after 4 weeks and were found to be reliable (Tables 8 and 9).

**Table 8:** Reliability of the retracing of different parameters with Bland- Altman method

Variables	Mean ± Standard deviation	95% Confidence Interval of the Difference		Difference
		Lower	Upper	
SNA	0.00 ± 1.017	−0.380	0.380	0.759
SNB	0.03 ± 0.99	−0.340	0.407	0.746
ANB	−0.06 ± 0.64	−0.306	0.172	0.477
Beta	0.26 ± 1.14	−0.160	0.693	0.853
W angle	−0.1 ± 1.15	−0.531	0.331	0.862
Length	0.00 ± 0.58	−0.219	0.219	0.438
AP diameter	0.00 ± 0.52	−0.196	0.196	0.392
Depth	−0.033 ± 0.615	−0.263	0.196	0.459

**Table 9:** Reliability of the retracing of shape, bridging and skeletal patterns

S.No.	Parameters	Kappa Value
1	Shape	1.000
2	Bridging	.939
3	Skeletal patterns	1.000

## Discussion

The study was carried to compare the shape, size, and bridging of sella turcica using lateral cephalogram in patients with different skeletal patterns and genders.

### Size of Sella turcica

The mean length, anteroposterior diameter and depth of sella turcica were 8.13 ± 2.03 mm, 9.60 ± 1.43 mm and 6.40 ± 1.21 mm respectively. When compared with this study the mean sella turcica size was larger in the studies done by Alkofide EA [9] Yassir YA et al. [25], Filipović G et al. [10], and Sathyanarayana HP et al. [11]. This may be attributed to the difference in ethnicity, genetic makeup, and environmental factors that may be present between the different populations, and to the methods of measurements in which the magnification found in the lateral cephalogram may not have been corrected.

When determining if any differences existed in the current study between males and females regarding the sella turcica size, no significant gender difference was found as in the study done by Alkofide EA [9]. Contrary to our results, Axelsson et al. [23] and Sathyanarayana HP et al. [11] found that there was a significant difference in lengths of sella between males and females. Pubertal growth spurt in females begins 2 years earlier than males, so a significant change in pituitary fossa size occurs in females from 11 to 15 years of age and the late growth acceleration in males, which is usually 2-3 years later than that in females which results in an approximate equalization in sella area in both genders [9]. The age of the patients in the study was 18-30 years in compared to Axelsson et al. [23] (6-21 years), Sathyanarayana HP et al. [11] (9-27 years).

There was no significant difference in size of the sella turcica with the age of the patient in this study. The increase in the linear dimensions of sella turcica with age was found by Sathyanarayana HP et al. [11], Andredaki M et al. [26], and Alkofide EA [9]. The difference in the results may be due to the difference in age groups of the patients in the studies; in this study the age group of the patients was 18-30 years compared to Sathyanarayana HP et al. [11] (9-27 years), Andredaki M et al. [26] (6-17 years), Alkofide EA [9] (10-26 years). According to Sperber [4] increase in the size of the sella turcica is due to resorption and deposition of posterior wall and floor and this takes place until 16 to 17 years of age.

The linear dimensions of sella turcica were significantly larger in Class III skeletal pattern in comparison to Class I and Class II. Alkofide EA [9], Filipović G et al. [10], Sathyanarayana HP et al. [11] found out that the anteroposterior diameter was larger in class III and smaller in class II skeletal pattern in patients of Saudi Arabia, Serbia, and South India respectively. Contrary to this, Meyer-Marcotty P et al. [14] and Shah AM et al. [27] found out that there was no significant difference in size of sella turcica when compared with different skeletal patterns. These variations of the result may be because this study had used Beta and W angle along with ANB angle to compensate for various limitations of ANB and Wits [21, 22, 28, 29] whereas only ANB and Wits were used in other studies [14, 27].

### Shape of Sella turcica

Sixty percent of the patient had normal sella morphology, and 40 percentage had different variations in the morphology of sella turcica in this study. Axelsson et al. [23], Alkofide EA [9] and Sathyanarayana HP et al. [11] described the shape of sella turcica in Norwegian, Saudi and South Indian patients where they found that 68, 67 and 61% respectively had normal sella morphology and 32, 33 and 39% respectively had variation in the morphology. The irregular shape of dorsum sella was most common in the patient population of Nepali origin (15%), and similar findings were seen in the studies done by Alkofide EA [9] (11.1%), Sathyanarayana HP et al. [11] (15%).

There was no significant difference in morphology of sella turcica between genders in this study. Similar results were reported by Sathyanarayana HP et al. [11] where 59% of males and 63% of females had normal sella morphology in South Indian patients.

### Bridging of Sella turcica

In this study, no calcification was seen in 65% of the patients, partial calcification was seen in 23.33% of the patients, and complete calcification was seen in 11.67% of the patients. According to the study done by Leonardi et al. [15], 56.4% had no calcification, 33.7% had partial calcification, and 9.9% had complete calcification in control group, and 23.5% had no calcification. Sunderswaran S and Nipun A [30] in the control group from South India found that the prevalence of the partial calcification of sella turcica in was 23.43% when Leonardi et al. [15] method was used which was similar to the findings of this study but when they adopted a second method in which the interclinoid distance rather than the length of sella turcica the prevalence of partial calcification was 17.18%. This result shows the difference in the method that has been used. Kogali S et al. [31] performed a cadaveric study of 112 dry adult skull bones for the presence of sella turcica bridging and found that 8.04% had sella turcica bridging. Relatively higher percentage of bridging (11.67%) may have been seen in this study as this was a radiographic study. The differences between direct anatomical studies and the radiographic studies have been attributed to superimposition of the overlapping clinoid processes in the lateral cephalograms [32].

In this study, 5% of Class I had complete sella bridging whereas 12.5% of Class II and 17.5% of Class III had complete sella bridging. Similar results were seen by Meyer-Marcotty P et al. [14] where they found out that 9.4% of class I had sella bridging and 16.8% of Class III had sella bridging. Abdel-Kader HM [12] reported that 4.83% of Sella bridge was found in the orthodontic group and 6.19 percentage of sella bridge was found in the orthognathic group in Saudi patients. Jones RM et al. [2] found that the incidence of bridging in the combined surgical orthodontic group compared with the orthodontics-only group was 16.7% and 7.3%, respectively in patients from Germany. It may suggest that the type of malocclusion appears to play a major role in the prevalence of sella bridges as cited by Abdel-Kader HM [12].

This study showed that there was an increase in the frequency of sella turcica bridging in Class II and Class III skeletal patterns compared to Class I skeletal pattern. The increased frequency of sella bridging was seen in Class II skeletal pattern was reported by Obayis K et al. [33].

It must be realized that the radiographic fusion may be due to the fusion of structures and not real bony fusion [32]. Calcification of diaphragma sellae, or ‘bridging’ of the sella, without clinical signs or symptoms, is considered a normal variant of the sella turcica [34] although various pathological processes can be associated with this calcification [15, 16], and as far as etiology is concerned with Sella bridge, it may be considered malformation from prenatal phase of life due to the complex embryology of the sphenoid bone [15, 32]. According to this theory; a sella turcica bridge should be considered a developmental anomaly as cited by Leonardi et al. [15].

There are few limitations of this study. The ethnic variation that is present in the patient population of Nepali origin has not been considered during this study. This study has been done in lateral cephalogram which is a two-dimensional picture. Hence we are not able to depict the true anatomical size, shape, and bridging of the sella turcica. Another pitfall of this study was that only there was only single observer evaluating and measuring the cephalograms. It limits the study to confer the conclusions on dimensions, shape, and bridging of sella turcica. Three-dimensional studies of the sella turcica or cadaveric anatomical study of the human skull would be more informative in this regard. Morphometry of sella turcica was not considered in this study which may require quantitative methods to measure shape and morphology of sella turcica. The evaluations described above are subjective and do not provide quantitative data.

## Conclusions

1. Sixty percent of the investigated subjects had a normal sella shape.
2. The mean length of sella turcica was 7.91 ± 1.52 mm in Class I, 7.32 ± 1.62 mm in Class II and 9.16 ± .2.42 in Class III skeletal pattern; anteroposterior diameter was 9.30 ± 1.02 mm in Class I, 9.15 ± 1.28 mm in Class II and 10.35 ± 1.64 mm in Class III skeletal pattern; and the depth was 6.40 ± 0.92 mm in Class I, 6.07 ± 1.01 mm in Class II and 6.74 ± .1.54 mm in Class III skeletal pattern.
3. There was no significant difference in size of sella turcica between genders and age (18-30 years).
4. A significant difference was found in length and anteroposterior diameter size between Class I and Class II and Class III patients. The larger size was present in skeletal Class III patients.
5. Complete calcification was seen in 11.67% of patients in this study. There was an increased prevalence of sella turcica bridging in Class III and Class II skeletal pattern compared to Class I skeletal pattern in the patients in this study but was found to be statistically insignificant.
6. The results of the present study of shape, size, and bridging of sella turcica may be used as reference standards in future for Nepali subjects when studying sella turcica morphology.

## Abbreviations

BPKIHS: Bishweshwar Prasad Koirala Institute of Health Sciences
CODS: College of Dental Surgery
DS: Dorsum Sella
N: Nasion
OPD: Out-Patient Department
Or: Orbitale
Po: Porion
Point A: Subspinale
Point B: Supramentale
S: Sella
SN: Sella-Nasion
